# Reply to “Evaluation of a commercial multiplex pathogen panel for the diagnosis of pediatric *Kingella kingae* joint infections”

**DOI:** 10.1128/jcm.01060-25

**Published:** 2025-09-03

**Authors:** Laura M. Filkins, Lawson Copley

**Affiliations:** 1 Department of Pathology, University of Texas Southwestern Medical Center 12334, Dallas, Texas, USA; 2 Department of Pediatric Orthopaedic Surgery, Children’s Medical Center of Dallas and University of Texas Southwestern Medical Center 12334, Dallas, Texas, USA; Cleveland Clinic, Cleveland, Ohio, USA

## REPLY

We appreciate the comments by P. Bidet et al. We agree that detecting *Kingella kingae* in joint fluids is an ongoing challenge and welcome the addition of their BIOFIRE Joint Infection (JI) panel versus King-UX PCR performance comparison data to the scientific literature. To date, comparison of *K. kingae* detection by the BIOFIRE JI Panel to other targeted PCR methods is limitedly reported. Targeted PCRs or 16S rRNA PCR followed by sequencing are often reported for discrepancy evaluation of additional positive detections by the BIOFIRE JI panel compared to culture and have shown high concordance with the BIOFIRE JI panel in these scenarios (1–3). The findings by P. Bidet and colleagues highlight that the additional nucleic acid detection methods may offer enhanced sensitivity for *K. kingae* compared to the BIOFIRE JI panel. Sanchez et al. similarly described lower detection of *K. kingae* by the BIOFIRE JI panel compared to their laboratory-developed PCR in joint specimens, though the difference in detecting *K. kingae* was more modest in this study, with a positive percent agreement of 93.8% (15/16) (4).

In our study, “Performance evaluation of a commercial multiplex pathogen panel for the diagnosis of pediatric joint infections,” we compared the performance of the BIOFIRE JI panel to conventional testing that was actively utilized at our pediatric institution, including body fluid culture with or without 16S rRNA PCR followed by Sanger sequencing, as ordered by the clinical provider (5). A limitation of this study design is that not all potential methods of detecting pathogens from joint fluids were performed on all specimens (e.g., anaerobic, mycobacterial, and fungal cultures; individual targeted PCRs for each common pathogen, including *K. kingae*; or metagenomic sequencing) and, therefore, it is not a global method comparison. However, this study design reflected the true practice at our institution and, hopefully, provides insight into the possible benefits (e.g., the potential to shorten the time to optimal antimicrobial therapy in 27.7% of patients in our study) and trade-offs (e.g., imperfect analytic and clinical performance of any one test, and inclusion of rarely detected targets, [1]) of utilizing a molecular panel like the BIOFIRE JI panel as part of the diagnostic approach to pediatric joint infections. We also note that the EDTA and sodium-heparin collection devices were not used in our study, and these are not currently approved for use with the BIOFIRE JI panel in the U.S.

The optimal approach to testing is one that maximizes the detection of clinically significant pathogens, supports prompt and optimized clinical management, including antimicrobial therapy, and avoids unnecessary testing or procedures. In addition to analytic test performance, test selection must consider and balance the strengths and limitations derived from the patient- and institution- or practice-specific factors. Patient factors may include age, type of joint infection, prior receipt of antimicrobial therapy, and other risk factors. Institution- or practice-specific factors can include which tests are available, time to results, cadence of adjusting therapy and empiric therapy coverage, local antibiogram patterns, available information systems tools, methods for managing specimen use, and payor mix and reimbursement determinations. Given the various factors, there is unlikely a single best approach for all. Importantly, patient care is optimized through collaboration between experts in clinical microbiology, orthopedics, infectious disease and antimicrobial stewardship, and other clinical stakeholders to guide appropriate testing that is supported by robust internal and external clinical data (6, 7).
